# The *European Journal of General Practice* provides open access for all. ‘Where are we, where are we going?’

**DOI:** 10.1080/13814788.2017.1291906

**Published:** 2017-02-24

**Authors:** Jelle Stoffers

**Affiliations:** ^a^ European Journal of General Practice, Department of Family Medicine, Care and Public Health Research Institute (CAPHRI), Maastricht UniversityNetherlands


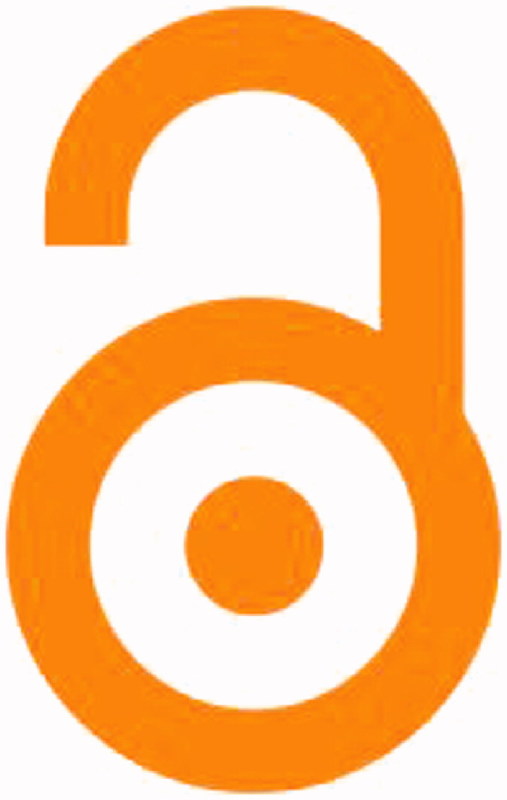


The subtitle of this paper refers to an Editorial by Fons Sips, who was the founding Editor-in-Chief of the *European Journal of General Practice* (EJGP) from 1995 to 1999 [1]. Last year, Fons Sips died at the age of seventy-five. Before I became the Editor-in-Chief of this journal, I only had met him occasionally. Later, at a Wonca Europe Conference, we had a short conversation on how the journal was doing. From the various obituaries that were published after Fons’s passing (e.g. www.woncaeurope.org/content/fons-sips-2016, http://vdgm.woncaeurope.org/content/memory-fons-sips, www.bmj.com/content/354/bmj.i4892), the picture emerges of a passionate general practitioner who developed into an international advocate for the cause of high-quality general practice/family medicine (GP/FM) across Europe. He was acknowledged and valued by many.

When you read his Editorials, it is clear that Sips was a promoter of patient-centred family medicine, delivered by personal doctors, who were trained by qualified teachers, using evidence-based knowledge, provided by general practice research. He regarded science to be an international means of bridging differences between cultures and healthcare systems and at the same time raising the status of general practice across Europe. Sips saw this journal as a unifying medium connecting the worlds of researchers, teachers, and practitioners of family medicine in Europe. Consequently, he wrote about ‘the European dimension’ as a criterion for acceptance of a manuscript to be published in this journal. Authors should be challenged to answer the question what their situation had in common with their European colleagues, or how their particular setting could inspire others working under different circumstances [1,2].

In his farewell editorial [3], Sips concluded that after four years of intensive collaboration between authors, editors, referees, the advisory board, European network organizations, national colleges and academies, the executive committee of Wonca Europe, and the publisher, the primary goal had been achieved: ‘the journal has survived the first most difficult years.’ At the time, the bid for indexation in *Index Medicus/Medline* – the ‘maturity test,’ Sips called it – was in progress. Sips acknowledged the constructive contributions of referees in the review process. He appreciated ‘their dedication to set up a quality standard for the journal.’ He saw their efforts as an illustration of ‘the European-wide involvement of general practitioners and family physicians in fostering GP/FM as a scientific discipline’ [3].

Reading these editorials, I felt reassured: we still act in the same spirit! The current team of EJGP editors shares the same passion for effectively communicating relevant research findings of high quality from and to our colleagues across Europe and beyond. We too rely heavily on the goodwill and the quality of our reviewers. And finally, I recognize how the ‘intensive collaboration’ of many colleagues – authors, editors, reviewers, the Wonca Europe Executive – and the publisher, continues to be an essential condition for this journal to flourish. Only by these joint efforts could the EJGP successfully adapt to changes in society (‘internet’) and the world of scientific publishing. Since 1999, this journal has passed not one but two ‘maturity tests’: its indexation in *Index Medicus/Medline* (‘PubMed’) in 2003 and in the *Science* *Citation Index Expanded* (and therefore its listing in the *Journal Citation Reports* with an ‘impact factor’) in 2012. The journal is published online since 2006, and from this year on, 2017, the journal will provide ‘gold’ open access to all its readers.

## Open access

I hope that many readers agree with me, that this is a historic step forward. In a previous Editorial, I have written about the inevitability of open access publishing, and about its opportunities and challenges [4]. I suggested that in family medicine, professional or scientific organizations could think of collective ways of providing ‘open access’ to their members. Wonca Europe responded positively to this challenge. Thanks to their generous investment, author groups with a corresponding author from a member country of Wonca Europe pay a reduced Article Publishing Charge (APC) of €400 for a paper of more than 1500 words (regular APC €1000), and €200 for articles of 1500 or fewer words (regular APC €500). This arrangement between Wonca Europe and the publisher is valid for the years 2017–2021. We assume that this relatively small fee is attractive to many authors who are looking for an open access, peer-reviewed medical journal on primary healthcare to publish their work.

The Wonca Europe executive, the publisher and the editors of this journal hope that researchers in the field of GP/FM will not only consider but also choose the EJGP as the medium for their message. Imagine that all colleagues in Europe (and the rest of the world) now can read and use your article, provided they have an internet connection. Your message is available to all colleagues, students, vocational trainees, teachers, fellow researchers and even interested patients; it is at their fingertips, on their smartphones, tablets, laptops and office computers. Moreover, you can bring your article to their attention using Twitter and other social media: it is merely one click away. Thus, we hope that the intended readership of the EJGP – colleagues, students, vocational trainees, teachers, fellow researchers and interested patients – will soon discover that for them there are no barriers anymore in viewing, downloading, reading, forwarding, and citing our full-text articles.

In the spirit of Fons Sips, the *European Journal of General Practice* now can try to achieve its ambition of becoming ‘the’ scientific medium connecting the worlds of researchers, teachers, and practitioners of family medicine in Europe. Together we can build that European community of ‘authors’ and ‘readers’ in general practice/family medicine. Submit! Tweet us! Like us! Cite us! Use us!

We look forward to a great twenty-third volume of the EJGP!
